# Encountering the Unusual: Anesthesia Mumps as a Rare Postoperative Complication in a Ventriculoperitoneal Shunt Surgery

**DOI:** 10.14740/jmc5332

**Published:** 2026-07-01

**Authors:** Rufina Ning, Abdul Naeem

**Affiliations:** a Schulich School of Medicine, Western University, London, Ontario N6A 3K7, Canada; b London Health Sciences Centre University Hospital, Clinical Neurological Sciences, London, Ontario, Canada

**Keywords:** Postoperative anesthesia care, Neuroimaging, Anesthesia mumps, Ventriculoperitoneal shunt, Acute sialadenitis

## Abstract

An 87-year-old man with a history of World Health Organization (WHO) grade IV glioblastoma resection developed hydrocephalus and underwent a ventriculoperitoneal (VP) shunt insertion as an American Society of Anesthesiologists (ASA) class 4E patient with a body mass index (BMI) of 24.3 and postoperatively was found to have acute bilateral parotid glands swelling after 2 h post-extubation. He became acutely unwell with sinus tachycardia, hypertension, and a drop in SpO_2_ to 82%. Primary interventions included oxygen supplementation, 1 L bolus of normal saline, pain control, and a non-contrast computed tomography of the head and neck to evaluate cause of swelling, which revealed subcutaneous edema without evidence of hematoma. Patient was weaned off oxygen and monitored over the next 48 h, and face and neck swelling resolved without any further interventions. To our knowledge, the occurrence of anesthesia mumps (AM) described as acute, transient bilateral parotid gland swelling without purulence, has not been documented in the setting of a VP shunt insertion. This case represents a novel contribution to the literature and expands our understanding of the clinical contexts in which AM may arise. Risk factors for AM in this case included poor oral hygiene and nutritional status, prolonged “nothing by mouth” (NPO) status while awaiting surgery, and significant rotation of the head in neurosurgery such as a VP shunt insertion.

## Introduction

Anesthesia mumps (AM) is a rare, rapidly emerging condition also known as acute transient postoperative sialadenitis characterized by the onset of unilateral or bilateral parotid gland swelling, which may exhibit tenderness upon palpation [1]. Notably, such swelling manifests without overt signs of infection emanating from the Stensen’s duct and typically resolves spontaneously within a timeframe ranging from several hours to days, without engendering permanent sequelae [1, 2]. Nevertheless, it is imperative to underscore that in a subset of severe manifestations, there exists a tangible risk of airway obstruction, necessitating meticulous monitoring.

The etiological spectrum of AM can be divided into three broad categories: patient, anesthesia, or surgical factors. Patient factors include obesity given large neck circumference [3, 4], history of parotid disease such as sialolithiasis or acute bacterial sialadenitis, or dehydration leading to the thickening of salivary secretions and potential blockage within the salivary glands [1]. Immunocompromised states leading to salivary stasis such as malnutrition, diabetes, Sjögren’s syndrome, and human immunodeficiency virus (HIV) have also been suspected as patient-specific susceptibility factors for AM [5]. Anesthesia-related factors include perioperative usage of vasopressors leading to sympathetic activation and decreased salivary flow [1, 6], increased positive pressure in the oral cavity due to strain against endotracheal intubation [7], inadvertent activation of the pharyngeal reflex resulting in increased vasodilation and hyperemia in the parotid gland [2], mechanical trauma during endotracheal intubation impacting the salivary glands, introduction of air causing pneumoparotitis (typically with air seen on imaging), adverse drug reactions (as reported with morphine) [8] or specific drug effects on the parotid gland, such as using anticholinergics for reversal [2, 5]. AM has been seen in multiple anesthesia modalities including laryngeal mask insertion [9], general [10], neuroaxial [11], and combined anesthesia [3]. Surgical factors include extended duration of patient head positioning [12], lateral decubitus [13], or steep Trendelenburg positioning [5], which may induce pressure ischemia or compromise the salivary glands’ vascular flow [7, 14], and long operation times [1]. AM has been seen in various procedures and surgical disciplines as well, including endoscopy [1], thoracic, otolaryngology, urology, orthopedics, obstetrics and gynecology, plastic surgery, and neurosurgery [15]. The etiology of AM is frequently reported as multifactorial and no meta-analysis has identified any specific risk factors to date, likely due to its low estimated incidence (0.16%) [2] and the predominantly retrospective nature of reported cases.

While the majority of AM instances are amenable to conservative management strategies—encompassing intravenous (IV) hydration, analgesia, massage, and the application of warm compresses [16]—there are exigent scenarios wherein re-intubation becomes critical such as pronounced glandular swelling that compromises the airway [8, 17]. The extension of the swelling can reach into the parapharyngeal space resulting in significant airway narrowing as seen in previous imaging [18]. Other severe manifestations of AM have also caused neurological complications, such as brachial plexopathy or temporary facial paralysis [16, 17]. Several preventative measures have been suggested to mitigate the risk of AM, including appropriate cushioning and padding around the head and neck, optimizing hydration status, and reducing endotracheal strain upon intubation and extubation [19]. However, no single measure has been shown in the literature to reliably prevent its occurrence, likely contributing to the elusive multifactorial etiology of AM.

In this case report, we discuss the first report of AM in a frequently performed neurological procedure, a ventriculoperitoneal (VP) shunt insertion, and discuss the factors that may have contributed to their presentation.

## Case Report

### Investigations

An 87-year-old man with a history of high-grade glioblastoma (World Health Organization (WHO) grade IV) resection developed hydrocephalus due to an entrapped temporal horn of the left lateral ventricle postoperatively. Unfortunately, he developed an expressive aphasia after the resection and his hydrocephalus resulted in periodic confusion, worsening cognition, and inconsistent food intake. His past medical history included atrial fibrillation, chronic obstructive pulmonary disease, diabetes, abdominal aortic aneurysm repair, and transient ischemic accidents with a history of stroke. The patient was initially admitted for glioblastoma resection and postoperative recovery, and by hospital day 40, the decision was made to pursue surgical management of his hydrocephalus for palliative management of symptoms. He underwent a VP shunt insertion as an ASA class 4E patient with a body mass index (BMI) of 24.3 kg/m^2^, with a Mallampati score of 2. Anesthesia was induced intravenously with 100 µg of fentanyl, 150 mg of propofol, and 50 mg of rocuronium. A grade 2 view of the cords was achieved with a MAC blade size 3 and a size 8 endotracheal tube was placed and secured at 23 cm. Two grams of cefazolin was given prior to initial surgical incision. Anesthetic maintenance was done with 2% sevoflurane and a 60 µg bolus of remifentanil was given for the pinning of the skull. The head was secured in a Mayfield three-pin head holder for optical frameless stereotactic neuronavigation, which was used to guide ventricular catheter placement and optimize catheter trajectory. The anesthesia record documented a total surgical duration of 135 min. Based on the intraoperative record, the patient’s head was rotated to the right for approximately 114 min, from final positioning until extubation. A curvilinear occipitoparietal incision was centered over Frazier’s point, approximately 6 cm above and 3–4 cm lateral to the inion, for posterior ventricular catheter insertion. At the end of the case, 100 mg of sugammadex IV was given for reversal of neuromuscular blockade. The surgical procedure was successfully completed without any complications and the patient was extubated in the operating room (OR) before being subsequently transferred to the post-anesthesia care unit (PACU) for 2 h with unremarkable vitals. At the PACU, he was given 0.4 mg of subcutaneous hydromorphone and 15 mg of IV ketorolac for pain.

Upon transfer to the surgical ward, there was significant swelling of the bilateral parotid glands and neck region noted, alarming staff after an otherwise stable PACU stay. Oral examination did not reveal any purulent exudate from the Stensen’s duct or swelling of the throat, but poor oral hygiene and tenderness were seen upon palpation of the parotid glands, with the right side more painful than the left. The patient developed sinus tachycardia (112 bpm), a blood pressure spike of 172/77 mm Hg, a respiratory rate of 14 breaths per minute, a temperature of 36.9 °C, and an SpO_2_ of 82%, but no significant respiratory distress was noted. He was not able to communicate with words regarding his symptoms, but neurologically appeared to be at baseline. Cardiac and respiratory physical exams were non-contributory.

### Diagnosis

Initial differential diagnosis included postoperative hematoma, pneumoparotitis, acute bacterial sialadenitis, sialolithiasis, and anaphylaxis. Preliminary laboratory workup revealed no significant complete blood count (CBC) changes from preoperative baseline, mild dehydration with hypernatremia (147 mmol/L) and hyperchloremia (110 mmol/L) on a CHEM-7, and no significant acid-base derangements on venous blood gas. After stabilizing the patient, he was sent for urgent computed tomography (CT) of the head and neck, which displayed a non-specific, diffuse enlargement of the parotid glands accompanied by subcutaneous edema, without evidence of hematoma (Fig. 1b). Submandibular and thyroid glands were within normal limits. These parotid gland enlargements were markedly notable when compared to prior preoperative imaging (Fig. 1a). The differential diagnosis at this point included parotid sialadenitis secondary to surgical positioning, infection such as viral mumps or suppurative parotitis, or rarer autoimmune or infiltrative etiologies such as sarcoidosis. Further laboratory workup came back with a negative mumps serology.

**Figure 1:**
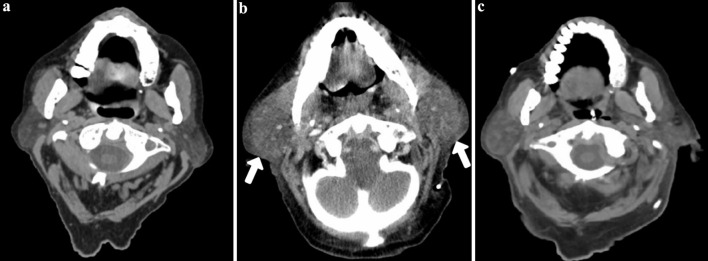
Computed tomography (CT) of the head and neck illustrating the progression and resolution of anesthesia mumps (bilateral parotid swelling). (a) Before the insertion of the ventriculoperitoneal (VP) shunt, the parotid glands are observed in their normal state. (b) Following the VP shunt procedure, bilateral enlargement of the parotid glands (white arrows) is evident. (c) Subsequent imaging, conducted within 48 h later, showed significant reduction in swelling, with the parotid glands nearly returning to their baseline condition.

### Treatment

Initial stabilizing resuscitation measures were performed, including oxygen supplementation at 3 L/min via nasal prongs, which improved SpO_2_ to 100%. His vitals subsequently returned to baseline after a 1 L bolus of normal saline and 0.5 mg of subcutaneous hydromorphone were administered to treat the dehydration seen on labs and pain, respectively. He was already on 2 mg of dexamethasone twice daily due to his glioblastoma and the dosage was not changed throughout his admission. The patient’s airway and his face and neck swelling were periodically monitored, as his neck circumference increased to 46.5 cm over the next 24 h from 41 cm at baseline but there appeared to be no compromise to the airway or increased respiratory distress at any point, ruling out the need for re-intubation.

### Follow-up and outcomes

After initiation of treatment for 24 h, we saw a notable amelioration in the bilateral parotid swelling and a follow-up CT scan within 48 h of initial presentation indicated a substantial reduction in the size of the parotid glands, virtually returning to baseline and a normal neck circumference of 41 cm (Fig. 1c). The patient was weaned off nasal prong oxygen after 48 h. The patient had no lingering side effects such as pain or enlargement of the parotid glands from the AM and was discharged several days later. Given that the initial bilateral parotid swelling occurred acutely during the postoperative period, the lack of purulent discharge from Stensen’s duct, and the swelling resolving within 48 h, the patient likely had AM precipitated by surgery and with risk factors associated with the parotid glands that primed an acute inflammatory response.

## Discussion

To our knowledge, based on a review of existing literature, the present case represents the first ever documentation of AM post-VP shunt placement, indicating that even a commonly performed neurosurgical procedure with a moderate surgery time and specific head positioning can precipitate AM. In this case report, several factors likely predisposed the patient to the emergence of AM. The patient’s characteristics including a prolonged hospital stay (post-admission day 40), depressed level of consciousness due to hydrocephalus, and awaiting surgery for 36 h while intermittently nothing by mouth (NPO) with poor food intake likely contributed to a deconditioned nutritional status, dehydration, and stasis of the salivary glands, on top of poor oral hygiene noted. Due to his glioblastoma, he was also started on 4 mg of dexamethasone twice daily for 8 days before continuing on 2 mg of dexamethasone twice daily for the past 40 days; notably long-term, systemic corticosteroid use has been seen to increase salivary viscosity [20] and may also contribute to salivary stasis. With these risk factors present [1], AM was likely then precipitated by the significant head rotation that remained rotated for nearly the entire surgical time during the VP shunt insertion surgery. Similarly, skull base surgeries requiring substantial head rotation for surgical access have precipitated incidences of AM [16, 21]. Direct mechanical trauma or compression of the parotid glands during VP catheter passage to the peritoneal cavity did not occur as the operating surgeon reported no significant resistance or compression while tunneling the catheter behind the ear.

While steroids and sialagogues are also commonly administered to manage acute inflammation in AM, additional antibiotic usage outside of standard postoperative prophylaxis should only be considered if the clinical presentation suggests a secondary bacterial infection within the salivary ducts consequent to reduced salivary gland flow [21]. Non-steroidal anti-inflammatory drugs (NSAIDs) can also be used for conservative treatment [11]. In our case, additional steroids, anti-inflammatories, and sialagogues were not given as the inflammation appeared to be reducing by 24 h and antibiotics were not indicated as no purulent discharge was seen from the parotid glands and CBC remained at baseline. He had also received 15 mg of ketorolac at PACU postoperatively, which may have contributed to alleviating his inflammation. Insights learned from a systematic review of skull-base surgeries emphasized the significance of ergonomic intraoperative positioning strategies to prevent excessive flexion and rotation of the patient’s head and neck and the importance of maintaining a low threshold for re-intubation to ensure a timely intervention to address impending airway compromise [21]. Future research should also evaluate whether AM incidence can be correlated with the number of predisposing risk factors, which may help identify higher-risk patient profiles.

The strengths in our approach included a multi-disciplinary team where neurosurgery, anesthesiology, radiology, critical care, and internal medicine were consulted on the patient to ensure that a broad differential diagnosis was evaluated and vigilant monitoring for airway involvement was maintained. The limitations to our approach include the lack of other supportive care measures as documented in the literature being applied to our patient, such as massages and warm compresses being applied. Given the limited institutional familiarity with AM, it also reduced awareness of implementing preventative measures, such as mitigating poor oral hygiene by evaluating nutritional status, stasis of salivary glands prior to surgery due to NPO status, or time spent with head rotation during surgery.

In summary, it is imperative for neurosurgeons and anesthesiologists to maintain vigilance for the occurrence of AM in postoperative neurosurgical patients, particularly those whose procedures necessitated substantial head rotation and involved a patient population prone to dehydration and compromised nutritional status. The management of AM in the PACU presents a significant challenge, characterized by the sudden onset of bilateral parotid gland swelling subsequent to surgeries that were technically seamless, compounded by the ensuing uncertainty, confusion, and the potential for acute airway obstruction. The primary management objectives in cases of AM should center on airway stabilization, re-establishment of adequate hydration levels, and mitigation of inflammation, with the overarching aim of preempting the severe, potentially lethal complications associated with this condition.

### Learning points

In patients requiring surgery with prolonged head rotation, risk factors for AM such as prolonged NPO duration or poor oral hygiene should be considered in the preoperative setting, especially in patient populations that are prone to dehydration and compromised nutritional status.

Treatment of AM generally involves supportive treatment such as IV fluids, pain control, warm compresses, and massages. However, a short course of anti-inflammatories such as steroids, NSAIDs, and sialagogues to help with salivary flow have also been previously used.

Despite the transient nature of AM, the risk of significant sequelae reported in other case studies, such as facial nerve injury and airway compromise, underscores the importance of early recognition of this condition and close airway monitoring postoperatively.

## Data Availability

Any inquiries regarding supporting data availability of this study should be directed to the corresponding author.

## Abbreviations

AM: anesthesia mumps
ASA: American Society of Anesthesiologists
CBC: complete blood count
CT: computed tomography
HIV: human immunodeficiency virus
IV: intravenous
NPO: nothing by mouth
NSAIDs: non-steroidal anti-inflammatory drugs
PACU: post-anesthesia care unit
VP: ventriculoperitoneal
WHO: World Health Organization
